# Nucleoside-lipid-based nanocarriers for methylene blue delivery: potential application as anti-malarial drug†

**DOI:** 10.1039/c9ra02576f

**Published:** 2019-06-17

**Authors:** Koffi Kowouvi, Bruno Alies, Mathieu Gendrot, Alexandra Gaubert, Gaelle Vacher, Karen Gaudin, Joel Mosnier, Bruno Pradines, Philippe Barthelemy, Luc Grislain, Pascal Millet

**Affiliations:** Univ. Bordeaux, U1212 INSERM–UMR 5320 CNRS, ARNA, ChemBioPharm 146 rue Léo Saignat F-33076 Bordeaux France bruno.alies@u-bordeaux.fr pascal.millet@u-bordeaux.fr; Unité de Parasitologie et Entomologie, Département Microbiologie et Maladies Infectieuses, Institut de Recherche Biomédicale des Armées Marseille France; Aix-Marseille Univ., IRD, SSA, AP-HM, VITROME Marseille France; IHU Méditerranée Infection Marseille France; Univ. Bordeaux 146 rue Léo Saignat F-33076 Bordeaux France; Centre National de Référence du Paludisme Marseille France

## Abstract

Nucleolipid supramolecular assemblies are promising Drug Delivery Systems (DDS), particularly for nucleic acids. Studies based on negatively and positively charged nucleolipids (diC16dT and DOTAU, respectively) demonstrated appropriate stability, safety, and purity profile to be used as DDS. Methylene Blue (MB) remains a good antimalarial drug candidate, and could be considered for the treatment of uncomplicated or severe malaria. However, the development of MB as an antimalarial drug has been hampered by a high dose regimen required to obtain a proper effect, and a short plasmatic half life. We demonstrated that nanoparticles formed by nucleolipid encapsulation of MB using diC16dT and DOTAU (MB-NPs) is an interesting approach to improve drug stability and delivery. MB-NPs displayed sizes, PDI, zeta values, and colloidal stability allowing a possible use in intravenous formulations. Nanoparticles partially protected MB from oxido-reduction reactions, thus preventing early degradation during storage, and allowing prolongated pharmacokinetic in plasma. MB-NPs' efficacy, tested *in vitro* on sensitive or multidrug resistant strains of *Plasmodium falciparum*, was statistically similar to MB alone, with a slightly lower IC_50_. This nucleolipid-based approach to protect drugs against degradation represents a new alternative tool to be considered for malaria treatment.

## Abstract 2

Nucleolipids protects methylene blue against reduction (induced by light and chemical reductants) and do not impair antimalarial activity.

## Graphical abstract

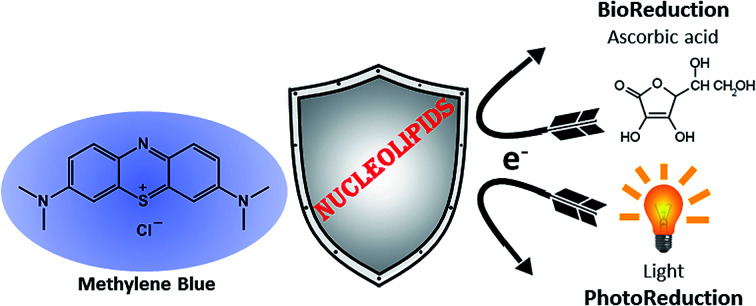

## 1. Introduction

Worldwide malaria eradication is hampered by *Plasmodium falciparum* resistance to new antimalarial drugs, including internationally recommended combination therapies based on artemisinin derivatives (ACT). The development of new antimalarial drugs is a mandatory step. However although many potential candidates have been identified, the high cost of drug development and the lack of R&D funding result in limited opportunities for new drugs to reach a clinical development status.^1^ Therefore, drugs that are already registered for other diseases and affordable in the public domain, represent an intermediate solution for the development of new antimalarial drug combination therapies.

**Fig. 1:**
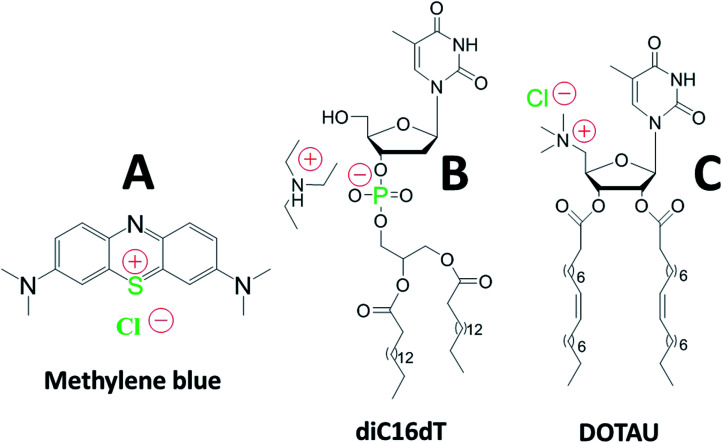
Chemical structures of methylene blue (A), anionic nucleotide-lipid diC16dT a thymidine 3′-(1,2-dipalmitoyl-*sn-glycero*-3-phosphate) (B), cationic-nucleoside-lipid DOTAU (2′,3′-dioleyl-5′-deoxy-5′-trimethyl-ammonium-uridine) (C). Green color highlight atoms identified with the EDX analysis (see Fig. 2c). Red color show molecules charges in solution.

Methylene Blue (MB) is a positively charged tricyclic phenothiazine molecule (Fig. 1) which has been used for malaria treatment.^2^

Paul Ehrlich highlighted in 1891 that dyes targeting some microorganisms and keeping the surrounding tissue unharmed could be used as drugs. MB especially presented a high affinity for *Plasmodium* parasites and low toxicity for the patients.^3^ However, in the early 1900's, quinine supplanted MB due to its efficacy at lower dose.^4^ Since then, MB has been approved for the treatment of methaemoglobinaemia, prevention of urinary tract infections, treatment and prevention of ifosfamide-induced neurotoxicity and intraoperative visualization of nerve tissues, endocrine glands, and fistulae.^5,6^

Recently, a new MB product was developed and approved by the authorities in Europe (2011) and the USA (2016) as therapeutic agent against methaemoglobinaemia. Proveblue® (Provepharm Life Solutions) contains limited organic impurities and heavy toxic metals; a great improvement compared to the other products on the market. Moreover, Proveblue® was more efficient than dihydroartemisinin for the prevention of cerebral malaria in a murine model.^7^

Up to now, clinical studies in humans and *in vitro* assays have revealed promising MB antimalarial activity either alone or in combined therapeutic approaches, against both asexual and sexual stages of *P. falciparum*.^8–12^ Additionally, MB exhibited high antimalarial activity against *P. falciparum* multiresistant strains and field isolates alone or in combination with dihydroartemisinin.^13–15^

However, the development of MB as an antimalarial drug has been hampered by a relatively high dose regimen required to be efficient. A dose around 15 mg kg^−1^ used in many clinical studies (about 1 g of MB per day for 60 kg weight during 3 days),^10,12,16,17^ led to a heavy oral administration in combination with other antimalarial drugs. Plasmatic bioavailability of MB rapidly reaches 85%, however the plasma half-life is less than 15 hours in adults,^18^ resulting in elevated and repeated drug administration to keep an appropriate plasmatic level and to obtain complete parasite elimination. The action mechanism of MB oxidized form (blue) is based on a non-competitive inhibition of glutathione reductase in the parasite cytosol.^5^ Still, its reduction by NADPH leads to the formation of leucomethylene blue (reduced form: colourless) decreasing MB efficiency. The protection of MB against reduction is a crucial point to enhance its plasma half-life and its action against the parasite.

An innovative approach to reach this goal is the development of nucleolipid based formulations.^19^ The use of negatively or positively charged nucleolipids (diC16dT and DOTAU respectively) previously demonstrated several advantages such as good stability and safety confirming their potentiality as Drug Delivery System (DDS).^20,21^ Formulation obtained by nanoprecipitation of these molecules allowed the formation of solid lipid nanoparticles (SLNs) with positive (SLN+) or negative (SLN−) charges depending on the nucleolipid used (Fig. 1).^22,23^ Based on this approach, we hypothesized that MB could (i) be protected against reduction and photodegradation, and (ii) present a higher affinity for infected red blood cells.

We report here the first example of MB-based SLNs stabilised by nucleolipids and their *in vitro* anti-malarial activity against *P. falciparum* parasite strains.

## 2. Materials and methods

### 2.1. Chemicals and reagents

MB (Proveblue®) was a gift from Provepharm Life Solutions (Marseilles, France). diC16dT (CAS number: 1160002-70-9) and DOTAU (CAS number: 868226-06-6) were synthesized in house according to previous publication.^20,21,24^ Chloroquine (CQ), quinine (QN), dihydroartemisinin (DHA) and doxycycline (DOX) were purchased from Sigma (Saint Louis, MO, USA). Amodiaquine (DQ) was provided by the WHO (Geneva, Switzerland) and mefloquine (MQ) was a gift from Roche (Paris, France). Lumefantrine (LMF) from Novartis Pharma (Basel, Switzerland), artesunate (AS), piperaquine (PPQ) and pyronaridine (PND) were purchased from Shin Poong Pharm Co. (Seoul, Korea). Absolute ethanol (EtOH) was purchased from VWR Chemicals (France). Water used in all experiments except *in vitro* experiment was produced in-house by ultrapure water system (ELGA Millipore system, minimum resistivity: 18.2 MΩ) and subsequently distilled to prevent any possible trace of resin from the ultrapure water system. MilliQ was purchased from Merck Millipore (MA, USA). RPMI (Roswell Park Memorial Institute) 1640 medium was purchased from Sigma Aldrich (France). Ascorbic acid and NaCl were purchased from COOPER (France). *P. falciparum* chloroquine-susceptible strains 3D7 (African) was obtained from MR4 (VA, USA) and multi-drug resistant W2 strain (Indochina) was obtained from MR4 (VA, USA).

### 2.2. Preparation of MB nanoparticles

#### MB/diC16dT nanoparticles (MB/diC16dT NPs)

A stock solution of diC16dT at 4 mg mL^−1^ prepared in EtOH was diluted in water (at 23 °C ± 1 °C) to get a resulting concentration of 0.48 mg mL^−1^. A stock solution of MB stock solution at 1 mg mL^−1^ prepared in EtOH was diluted in water (at 23 °C ± 1 °C) to get a resulting concentration of 0.17 mg mL^−1^. Similar volume of diluted solution of diC16dT (2838 μL) and MB (3000 μL) were mixed and vortexed (for 10 seconds at 1500 rpm) then stored in the dark at room temperature for 72 h.

#### MB/diC16dT/DOTAU nanoparticles (MB-NPs)

A lipidic film of DOTAU (0.24 mg) obtained from ethanol evaporation was rehydrated using MB/diC16dT NPs solution for 1 h at 37 °C under stirring (at 150 rpm). Final concentrations are 0.086, 0.23 and 0.041 mg mL^−1^ for MB, diC16dT and DOTAU, respectively.

### 2.3. Particle size and zeta potential measurements

Particle size and zeta potential analysis were performed at 25 °C using a Zetasizer Nano ZS (Malvern Panalytical, Orsay, France). Samples were diluted by 20 fold in distilled water, and measurements were performed at 25 °C. Mean diameter and polydispersity index (PDI) were measured in low volume disposable cuvettes whereas zeta potential were obtained using a folded capillary zeta cells. All analysis were performed in triplicate.

### 2.4. HPLC composition analysis

MB-NPs were analysed by HPLC on C_18_ column (YMC ODS-AQ 3 μM 4.0 × 50 mm) with mobile phase 60/40, methanol/H_2_O, v/v (both containing 20 mM ammonium acetate) during 3 min, then 95/5, methanol/H_2_O. Retention times were 1.3, 10 and 11.8 min for MB, diC16dT and DOTAU, respectively. Composition analysis was based on three calibration curve for each molecule.

### 2.5. Encapsulation efficiency

Encapsulation efficiency (EE) was determined using a direct method. 1 mL of samples was ultra-centrifuged at 14 100*g* for 20 minutes. Precipitate was diluted by 10 fold in EtOH and sonicated in an ultrasound bath at 50 °C for 20 minutes. The solutions were then analysed in triplicate using a UV-Visible spectrophotometer (Jasco V630). The acquisition parameters were: spectral range from 800 to 200 nm with a scan rate of 400 nm min^−1^, absorbance measurement at 665 nm for quantification and quartz cuvettes.
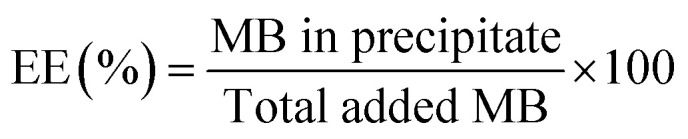


### 2.6. Transmission electronic microscopy and energy dispersive X-ray spectroscopy (TEM and EDX)

6 μL of NPs were loaded on a carbon-coated copper grid for 8 min before drying. The samples were observed using a Hitachi H-7650 electron microscope (Tokyo, Japan). Energy-dispersive X-ray spectroscopy was performed using a TECNAI transmission electron microscope (Thermo Fisher Scientific, USA) coupled with Quantax-X-Flash SVE 6.

### 2.7. FT-IR spectroscopic study

Spectra of MB, diC16dT, DOTAU and MB-NPs were acquired with a Fourier Transform Infrared spectrometer (FT-IR) (UATR spectrum two, PerkinElmer, France) controlled by Spectrum software. Samples were placed on the ATR module and crushed to ensure a homogeneous distribution on the crystal. All spectra were recorded by averaging 8 accumulations with 8 cm^−1^ resolution between 4000 and 450 cm^−1^. All spectra were obtained in the transmittance mode.

### 2.8. Stability studies

#### Colloidal stability in NaCl and RPMI

To assess MB/diC16dT NPs and MB-NPs stability in *in vitro* culture medium, a colloidal stability in NaCl and RPMI was performed. MB/diC16dT NPs and MB-NPs were diluted in a NaCl solution (62 mM of NaCl) and in RPMI media. The colloidal stability was assessed based on the size and PDI.

#### Colloidal stability at 37 °C

MB-NPs were stored at 37 °C in the dark for 28 days. Particle size and PDI modifications were monitored as previously described in triplicate.

#### Bio-reduction assessment

MB-NPs and MB solution (formulated in the same conditions as MB-NPs without nucleolipids) were diluted by 10 fold in distilled water. Ascorbic acid solution (5 mg mL^−1^ final concentration) was added to 1.9 mL of diluted MB-NPs and MB solution. At this addition of ascorbic acid, bio-reduction kinetics were monitored in visible at 665 nm for 15 minutes by a UV-Vis spectrophotometer (Jasco V630). The percentage of remaining MB is based on absorbance at 665 nm and normalized on initial absorbance. All measurements were done in triplicate.

#### Photosensitivity assessment

MB-NPs and MB solution were diluted by 10 fold in distilled water. Each solution was exposed to visible light (18 watts) for 42 h. The absorption intensity was monitored at 0, 1, 3, 5, 8, 12, 17, 21, 37 and 42 h of exposure. At each time point, the absorbance value was compared at the initial absorption. Analyses were done in triplicate.

### 2.9. *In vitro* experiments

Parasites susceptibility to the different MB formulations compared to other anti-malarial drugs was assessed on referenced strains of *Plasmodium falciparum*: 3D7 and W2. The Indochina clone W2 is representative of the resistant strains of *P. falciparum*. Clone W2 is multi-drug resistant, including pyrimethamine, CQ and QN. Clone 3D7 is susceptible to CQ and QN but resistant to MQ. The resistance traits of both clones were acquired naturally in the field and remain stable under culture conditions in the laboratory.^25^

A total of 100 μL of parasitized erythrocytes (final parasitaemia at 0.5% and a final haematocrit at 1.5%) was aliquoted into pre-dosed 96-well plates used for standard antimalarial drugs: CQ, QN, MQ, DQ, LMF, DHA, AS, PPQ, PND and DOX as previously described and in extemporaneous aliquoted plates for the different MB formulations.^26^ The plates were incubated for 72 h under controlled atmosphere at 85% N_2_, 10% O_2_, 5% CO_2_ and 37 °C. The drug susceptibility assay was performed using the *P. falciparum* drug sensitivity assay SYBR green I procedure from WWARN, adapted to our experiment.^27^

All antimalarial drugs were first dissolved in methanol and then diluted in MilliQ water to reach final concentrations that ranged from 6 to 3149 nM for QN; from 1.9 to 1988 nM for DQ; from 1.5 to 392 nM for MQ; from 0.1 to 107 nM for DHA and AS; from 1.9 to 998 nM for PPQ and from 0.5 to 497 μM for DOX. CQ and PND were diluted in MilliQ water for final concentrations ranging from 6 to 3231 nM and from 0.4 to 199 nM, respectively. LMF was diluted in EtOH to obtain final concentrations ranging from 0.6 to 310 nM.

MB was dissolved in distilled water to obtain MB solution. MB and MB-NPs solutions were then diluted in distilled water to final concentrations of MB ranging from 2 nM to 125 nM. For the photosensitivity assessment, MB and MB-NPs solutions were exposed to visible light for 16 h then were diluted under the same conditions used for the MB solution without light exposure ranging from 2 nM to 125 nM. For the bio-reduction assessment, distilled water was replaced by ascorbic acid solution (0.1 mg mL^−1^) to obtain the final concentrations of MB and MB-NPs.

Each batch of pre-dosed plates was validated using the CQ-resistant W2 strain in four independent experiments using conditions described below. The mean 50% inhibitory concentration (IC_50_) values for the CQ-resistant W2 strain and the different batches used over 3 years were 484 ± 40 nM for CQ, 388 ± 29 nM for QN, 97 ± 18 nM for DQ, 1.0 ± 0.4 nM for LMF, 26.3 ± 3.1 nM for MQ, 54.1 ± 5.4 nM for PPQ, 20.4 ± 3.4 nM for PND, 2.5 ± 0.4 nM for DHA, 1.5 ± 0.3 nM for AS, and 11.5 ± 1.9 μM for DOX. The polymorphic genetic markers msp1 and msp2 and microsatellite markers specific to *P. falciparum* were genotyped at least once a month to assess W2 clonality.^28,29^

All experiments were done in triplicate.

### 2.10. Statistical analysis of data

Data were statistically compared using the Student *t*-test (XLSTAT 2018, Addinsoft, France). IC_50_ were calculated using the following formula:
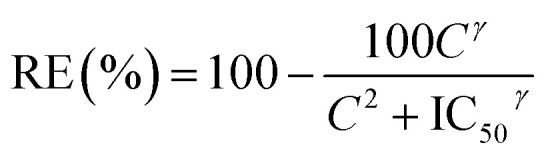
where: “RE” is the relative effect of the parasite in %; “*C*” is the concentration of the tested drug; “IC_50_” is the drug concentration inhibiting 50% of parasite's activity; “*γ*” is sigmoidicity factor which expresses the steepness of the curve.^30,31^ A difference between two groups or samples was considered significant with *p* < 0.05.

## 3. Results and discussion

### 3.1. NPs formulation and characterisation

**Fig. 2:**
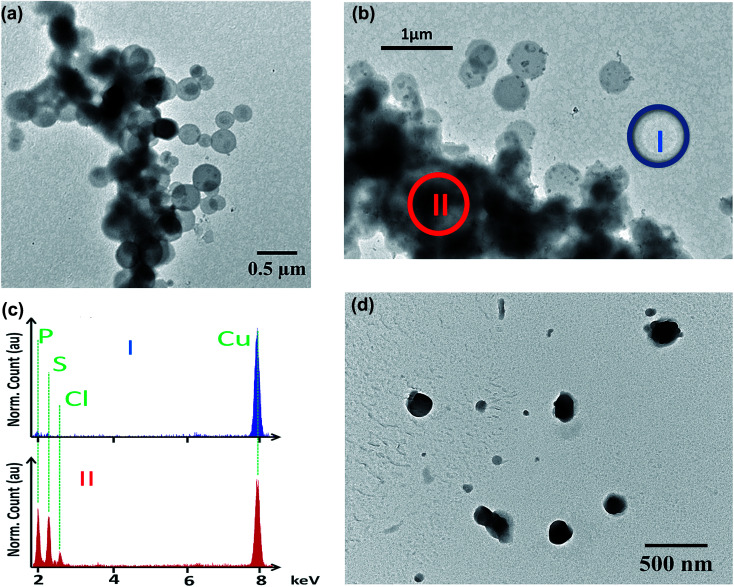
(a) TEM image of MB/diC16dT NPs. (b) TEM image of MB/diC16dT NPs with EDX acquisition analysis outside (I, blue) or on nanoparticles (II, red). (c) EDX spectra at positions I & II. Green dashed lines emphasized the emission of phosphor (corresponding to diC16dT), sulfur and chlorine (corresponding to MB). Both spectra were normalized with copper atom emission at 8 keV (due to TEM copper grid). (d) TEM image of MB-NPs.

MB is a tricyclic positively charged molecule. To formulate this active pharmaceutical ingredient as a SLN, the first step was to nanoprecipitate MB with diC16dT, a negatively charged nucleolipid.^20,22,23^ The association of these two compounds formed spherical nano-objects (Fig. 2a) with a diameter of 196.5 ± 4.2 nm measured by dynamic light scattering. This mean diameter was corroborated by TEM where particles size was around 200 nm (Fig. 2a). To check integrity of the nanoparticles observed in TEM, we performed EDX analysis (Fig. 2b). Phosphor (from diC16dT), sulfur and chlorine atoms (from MB) were present and co-localised confirming the composition of the nanoparticles (Fig. 2c). Nanoparticle population was monodispersed with a PDI of 0.078 ± 0.0078 and negatively charged with a zeta potential of −39.2 ± 2.7 mV. The electrostatic forces enabled NPs repulsion for at least 2 weeks, acting as indicators of formulation's stability. However, in media containing ionic species such as NaCl (62 mM) or RPMI media, MB/diC16dT NPs were destabilized (size < 9 nm and PDI > 0.4) due to a competition between MB, nucleolipid and charged species in the medium (Fig. S1 and S2†).

To overcome this drawback, a stabilization of the NPs by the addition of DOTAU (molar ratio 4 : 1, diC16dT/DOTAU), a positively charged nucleolipid, was tested.^20,22^ As for MB/diC16dT NPs, monodispersed nanoparticles were observed in distilled water (PDI: 0.08 ± 0.0086) by dynamic light scattering. The size was decreased by 26.6 nm (169.9 ± 6 nm) ensuring the potential use of this DDS by parenteral route.^32,33^ This mean diameter was consistent with TEM observations where particles size was around 180 nm (Fig. 2d). The addition of DOTAU did not impact the zeta potential (−34.87 ± 1.38 mV) indicating a reorganisation of the nucleolipids at the SLN external surface. In ionic media, the dilution of MB-NPs did not affect their size even if the dispersity was slightly increased (PDI of 0.227 ± 0.037 and 0.357 ± 0.021 in NaCl (62 mM) and RPMI media respectively). The combination of diC16dT and DOTAU improved colloidal stability in ionic media, indicating a stabilization of the structure (Fig. S1 and S2†).

**Fig. 3:**
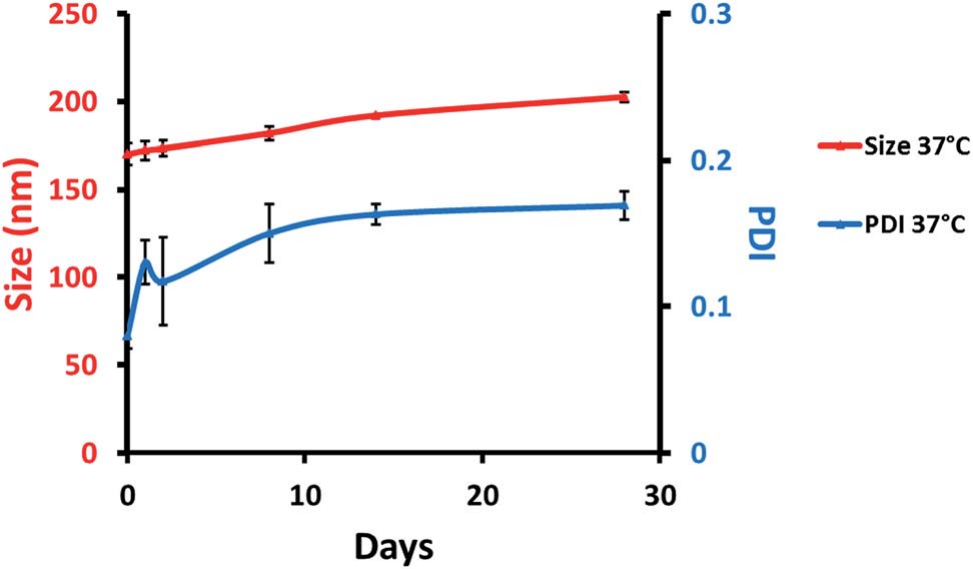
Colloidal stability of MB-NPs over time with particles size (red, left scale) and PDI (blue, right scale) at 37 °C. The errors represent standard deviation of three independent experiments.

Colloidal stability (size and PDI) of MB-NPs was evaluated at 37 °C using DLS (Fig. 3). NPs size and PDI remained stable, respectively under 200 nm and 0.2 at 37 °C allowing a potential use for *in vitro* and *in vivo* assays.

**Fig. 4:**
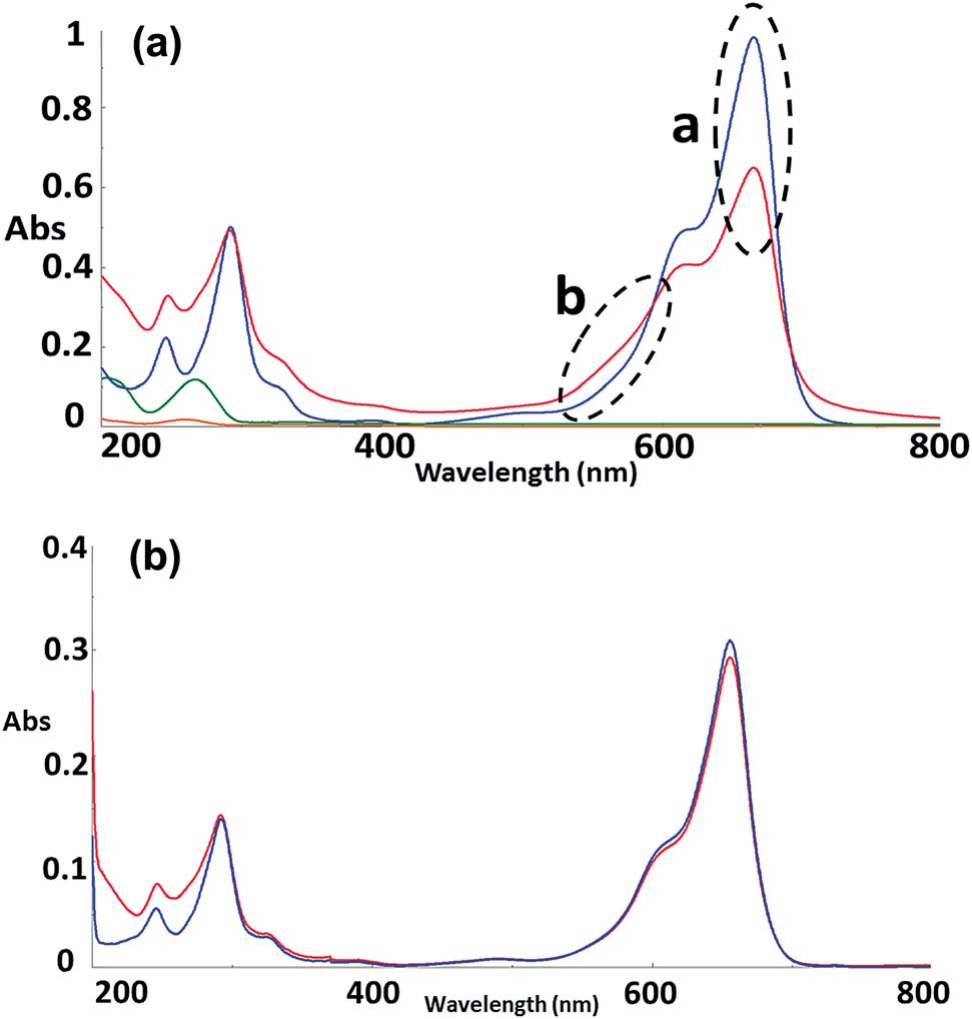
(a) UV-Visible spectra in water of MB (blue), diC16dT (green), DOTAU (orange) and MB-NPs (red). “a” and “b” spectra variations. *λ*_max_ = 665 nm. (b) UV-Visible spectra of MB solution (blue) and MB-NPs (red) solution after sonication in EtOH. *λ*_max_ = 655 nm.

**Fig. 5:**
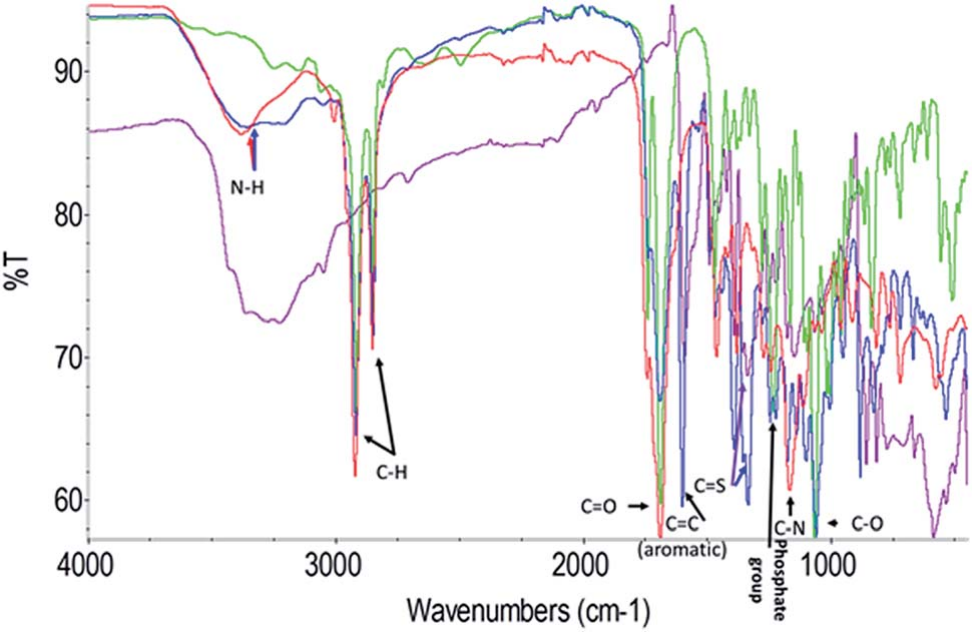
Fourier-transform spectra infrared spectra of MB (violet), diC16dT (green), DOTAU (red) and MB-NPs (blue). A zoom of the region 2000–1000 wavenumbers is shown in Fig S3.†

To further explore the organisation of the three compounds (MB, diC16dT and DOTAU) in the DDS, spectroscopic analyses were performed. Based on the UV-visible spectra (Fig. 4a), MB solution and MB nanoformulation were discriminated by the presence of an absorbance shoulder between 500 and 600 nm, a lower molar attenuation coefficient for MB-NPs. This change highlights the interaction between the three compounds stabilising the nano-object. Interactions involved are likely π-stacking, H-bonds, ionic as such change in UV-Vis spectra have been previously observed in presence of supramolecular assembly with MB,^34^ and nucleolipids.^35,36^ However, these interactions were disrupted by the addition of EtOH and sonication (50 °C for 20 min) (Fig. 4b) since no shoulder or lower absorbance were observed on the spectrum. In this condition, MB solution and MB-NPs are nearly identical in 500–700 nm visible region (blue *vs.* red trace, Fig. 4b). Loose interactions were confirmed by IR study (Fig. 5 and Table 1). MB, diC16dT and DOTAU characteristic bands were identified confirming MB-NPs composition, consistent with ionic interactions in the SLN.

**Table 1:** Chemical bond wavenumbers of MB, diC16dT, DOTAU and MB-NPs

Chemical bond (cm^−1^)	MB	diC16dT	DOTAU	MB-NPs
N–H	N/A	N/A	3380	3370
C–H	N/A	2916	2919	2920
		2850	2854	2850
C=O	N/A	1688	1692	1693
(Aromatic) C=C	1593	N/A	N/A	1601
C=S	1337	N/A	N/A	1334
Phosphate group	N/A	1231	N/A	1246
C–N	N/A	N/A	1166	1175
C–O	N/A	1066	1111	1061

MB-NPs composition was analysed by HPLC. MB-NPs is made of MB, diC16dT and DOTAU with molar proportion of 5, 4 and 1, respectively. The different amounts found suggest that the core of the MB-NPs is made of MB and diC16dT whereas DOTAU is likely involved in the coating of the MB-NPs.

The encapsulation efficiency was estimated at 86.7 ± 1.33% and 79.3 ± 0.97% for MB/diC16dT NPs and MB-NPs respectively. This difference was due to the competition between DOTAU and MB, both positively charged, leading to a partial release of MB in the medium.

### 3.2. Nucleolipid approach for MB protection

**Fig. 6:**
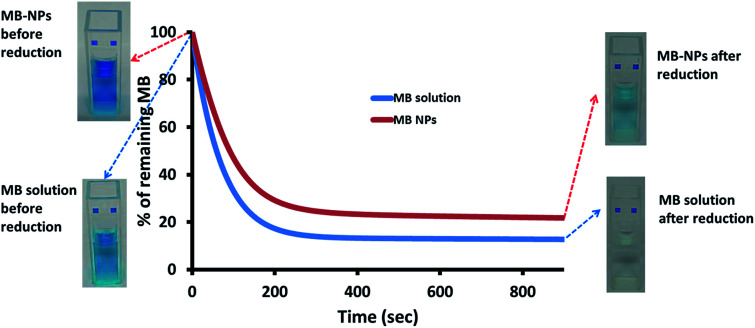
Reduction kinetics of MB solution (blue) and MB-NPs (red) in presence of ascorbic acid based on visible absorption at 665 nm. The widths of the curve represent standard deviation of three independent experiments.

MB is known for its sensitivity to oxido-reduction leading to the formation of leucomethylene. To evaluate the impact of encapsulation on this aspect, a bio-reduction assessment was performed. Ascorbic acid was added to MB and MB-NPs solutions. The reduction kinetics and solutions colour were monitored (Fig. 6). The absorption kinetics of MB solution was 1.4 fold faster than the one of MB-NP solution with a reduction up to 13% after only 15 min (colourless solution). For MB-NPs solution, 22% of MB was preserved after 15 min of exposition to ascorbic acid (blue solution). Therefore formulations of MB with nucleolipids (diC16dT and DOTAU) provide protection against reduction.

**Fig. 7:**
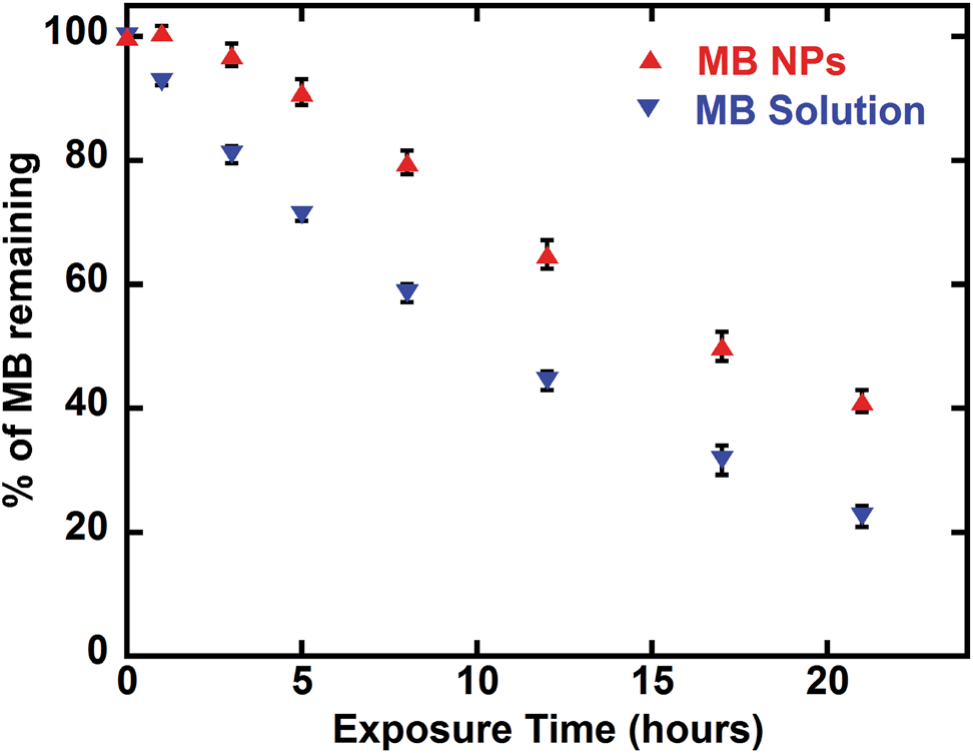
Photosensitivity of MB solution (blue down-pointing triangle) and MB-NPs (red up-pointing triangle) over time based on visible absorption at 665 nm. Error bars represent standard deviation of three independent experiments.

Another advantage of drug encapsulation is the photo-protection of sensitive molecules.^37^ A photosensitivity assessment was performed exposing MB and MB-NPs solutions to light for 42 h. As shown in Fig. 7, MB content decreased 1.6 fold faster for MB solutions compared to MB-NP samples (Student test, *p* < 0.05). The exposure time required to photobleach half of MB is faster (1.6 fold) in case of MB solution compared to MB-NP (10.5 h *vs.* 16.6 h, respectively). The association of nucleolipids with MB proved its partial protection against photosensitivity.

This approach represents a potentially new tool to improve MB stability (light and reduction), extend its half-life and thus increase its anti-malarial effect. This last point was explored by *in vitro* assays.

### 3.3. *In vitro* activity assessment

*In vitro* activity of MB-NPs were tested against the 3D7 *P. falciparum* (MQ resistant) or the W2 (CQ resistant and with QN reduced susceptibility) strains and compared to MB solution and standard antimalarial drugs. No significant differences were observed (*p* value > 0.05) and IC_50_ for all drugs were in the expected range against each strain (Table 2). IC_50_ of MB solution was 2 times higher against W2, compared to IC_50_ of 3D7 strain. These IC_50_ complied with usual IC_50_ obtained from isolates collected from imported malaria to France (mean IC_50_ of 15 nM for more than 600 isolates, personal data). In the case of MB-NPs, IC_50_ values were not statistically different from MB solution even if they were systematically lower. The nanoformulation concept did not alter the drug activity and even suggested a potential improvement of MB efficiency. To further investigate the interest of NP *in vitro*, light exposition impact on MB efficiency would be studied. Indeed, as previously highlighted, the nanoformulation of MB as SLN enabled a slower degradation. However, on a biological level, MB (solution or NPs) efficiency was characterized by higher IC_50_ values. MB in its oxidized form is taken up by the infected erythrocyte.^38^ Ascorbic acid was used to change MB upon reduction to leucomethylene blue and to prevent entry of MB into the red blood cell. The antiplasmodial activity of MB in combination with ascorbic acid should be lower. However, our present data showed that MB in combination with low concentrations of ascorbic acid was as active as MB used alone against the CQ-susceptible 3D7 strain (5.12 nM *versus* 5.23 nM) and more active against the CQ-resistant parasites W2 (6.61 nM *versus* 12.56 nM, *p* < 0.0001), suggesting a potentiation of the antimalarial effect of the BM. The nanoformulation maintained this ability of potentiation. When used alone at the same low concentration, ascorbic acid had no effect on *P. falciparum* parasites but at high concentrations (above 100 μM), ascorbic acid improved the *in vitro* growth of *P. falciparum* parasites and antagonized the effects of some antimalarial drugs like artesunate and doxycycline, due to its properties as antioxidant and free radical scavenger.^39^ New potential applications may be considered for the treatment of resistant strains by this approach.

**Table 2:** Chemosusceptibility of the strains to MB solution MB-NPs with or without exposure to light or ascorbic acid expressed with mean half maximal inhibitory concentration (IC_50_), standard deviation (SD) and confidence interval at 95% (CI)

			Drug (nM)					
			MB	MB-NPs	MB + light	MB-NPs + light	MB + Asc	MB-NP + Asc
Strain	3D7 *n* = 17	Mean IC_50_	5.23	3.67	15.9^a^	24.2^a^	5.12	7.93
		SD	2.18	1.93	9.16	5.43	3.19	1.87
		CI	[4.09–6.38]	[2.69–4.65]	[11.09–20.69]	[21.33–27.02]	[3.55–6.7]	[6.82–9.04]
	W2 *n* = 15	Mean IC_50_	12.56	9.38	26.50^a^	21.49^a^	6.61^a^	6.88^a^
		SD	2.39	5.11	9.13	6.3	1.38	1.8
		CI	[11.38–13.73]	[6.87–11.89]	[22.02–31]	[18.40–24.58]	[5.93–7.29]	[5.86–7.90]

a *p* value < 0.0001 *versus* MB.

## 4. Conclusion

This study is based on the hypothesis that a nanoformulation of Methylene Blue (MB) may have several advantages (such as IC_50_ and stability improvement) compared to MB alone. To test this hypothesis, we develop a novel nanoformulation based on the association of MB with 2 nucleolipids, diC16dT and DOTAU. This assembly led to the formation of colloidal stable nanoparticles (MB-NPs) even in complex media; a promising result for MB-NPs stability in plasma. MB-NPs based on multimodal interaction (ionic, π-stacking, H-bonding) proved its efficiency to protect MB from redox phenomena due to chemical compounds or light. Higher storage stability and extending half-life could be expected by the use of this approach. Besides the fact that the nanoformulation did not alter MB activity, lower IC_50_ values were observed for drug sensitive and resistant *P. falciparum* strains *in vitro*. Further *in vivo* investigations could be of great interest using the nucleolipid-based nanoparticles approach for drug protection, plasmatic half-life extension and potential synergistic effect, especially to overcome parasite resistance.

## Contribution

KK overall study design and experiments, manuscript writing; BA nanoparticles study design and experiments, manuscript writing; MG *in vitro* study design and experiments; AG nanoparticles study experiments and manuscript writing; GV study design and manuscript writing; KG study design; JM *in vitro* study design and experiment; BP *in vitro* study design; PB study design; LG study design; PM overall study design and manuscript writing.

## Conflicts of interest

There are no conflicts to declare.

## Supplementary Material

RA-009-C9RA02576F-s001
