# Supplementary material for: Nucleoside-lipid-based nanocarriers for methylene blue delivery: potential application as anti-malarial drug
Source: RSC Adv. 2019 Jun 17;9(33):18844–52. doi: 10.1039/c9ra02576f (PMC9064961; doi:10.1039/c9ra02576f)
Supplement: RA-009-C9RA02576F-s001 [file RA-009-C9RA02576F-s001.pdf]

Electronic Supplementary Material (ESI) for RSC Advances.
This journal is © The Royal Society of Chemistry 2019

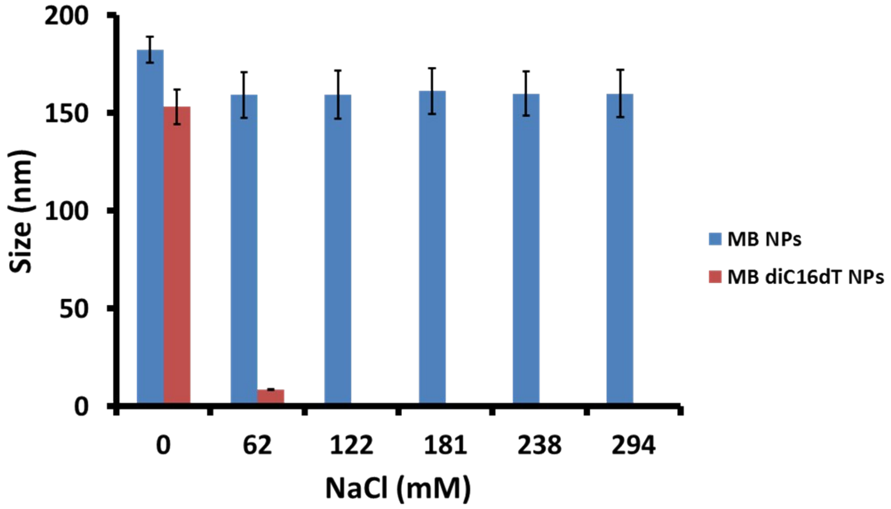


**Figure S1:** Size evolution of MB/diC16dT NPs (red) and MB-NPs (blue) at different concentrations of NaCl. Error bars represent standard deviation of three independents experiments.

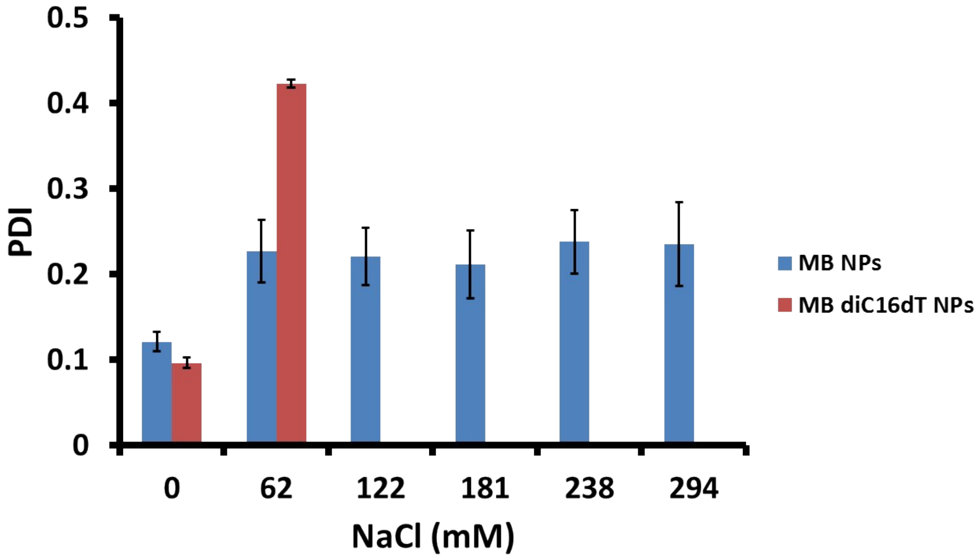


**Figure S2:** PDI evolution of MB/diC16dT (red) NPs and MB-NPs (blue) at different concentrations of NaCl. Error bars represent standard deviation of three independents experiments.

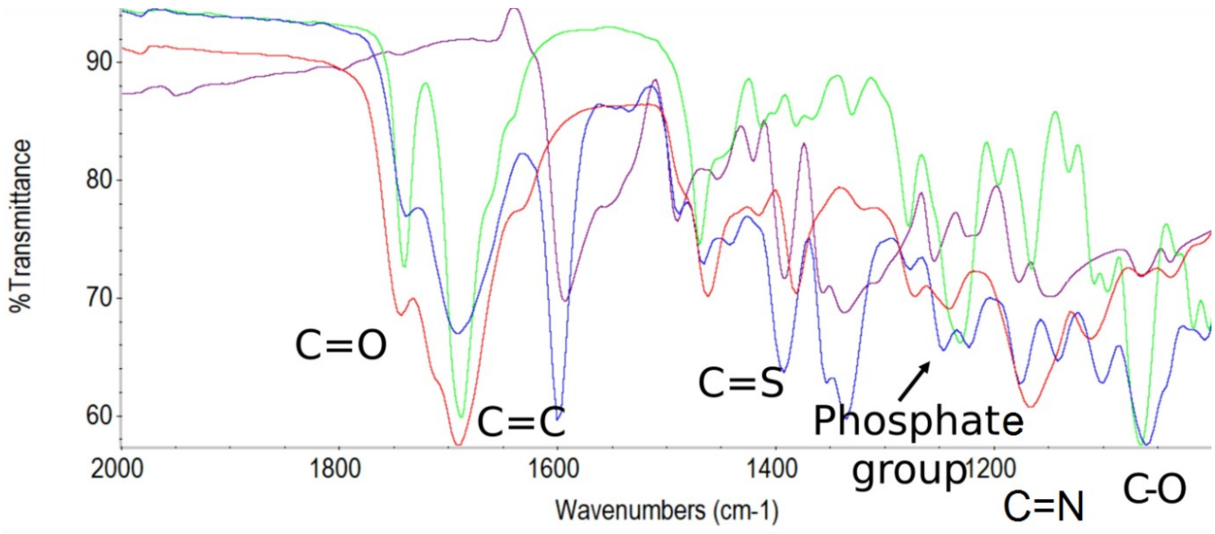


**Figure S3:** Enhancement of 2000-1000 Wavenumber region of Fourier-Transform spectra Infrared spectra of MB (violet), diC16dT (green), DOTAU (red) and MB-NPs (blue).
